# Pharmacology and Therapeutics in 1888

**Published:** 1889-02

**Authors:** 


### EDITORIAL.

### PHARMACOLOGY AND THERAPEUTICS IN 1888.

One of the best methods of self-instruction is to look back over a certain portion of the field covered by our work, and review what has been done, and see what mistakes have been corrected, what ideas shown to be true or false, and what real progress made. While the actual work in pharmacology and therapeutics in 1888 covered but comparatively few subjects, much progress was made, and a very large number of papers was published. The majority of these were not of great interest or value; but one that is at all conversant with medical literature knows that the chaff is always far in excess of the wheat. The chief work done in pharmacology during 1888 was in the field of organic compounds —a field that now seems to promise more than any other. The antiseptics and antipyretics belonging to the aromatic series have had a large share of attention, and the new hypnotics, belonging to the alcoholic series, have been investigated. Therapeutics proper has had less attention, is unfortunately still widely separated from pharmacology, and is largely empirical.

One of the few bodies belonging to the organic series of compounds recently introduced into therapeutics which has not only held its place but has gone on increasing in usefulness is antipyrin. In the early part of 1888 Savadovski published an exhaustive paper on this drug, the result of a series of experiments conducted under the direction of Professor Botkin. He investigated the action of the drug on the circulation, respiration, digestion, and the nervous system; on the nitrogenous change; on temperature, and on decomposition and fermentation. The acceleration of the pulse was found to be due to excitation of the excito-motor ganglia of the heart as it occurs after division of the vagus nerves and of the spinal cord. The blood-pressure was raised by the increased cardiac activity, and not by the effect on the peripheral arteries; in fact, the passage of blood or serum containing a certain amount of antipyrin through living vessels caused dilatation of the vessels. In regard to the influence of the drug on the chemical changes in the blood, it was found that oxyhæmoglobin solution mixed with 5 per centum of antipyrin remained unchanged for twelve hours. But in blood 2 per centum of antipyrin caused destruction of the red corpuscles in two or three hours, while 3 to 5 per centum causes a laky condition of the blood in from one and a half to two hours. Antipyrin does not affect the oxygen change in the blood. Savadovski found that antipyrin has no effect on the nitrogenous change in afebrile conditions, but that it produces an increased nitrogenous excretion in febrile dogs.

Lépine and Porteret have investigated the effect of antipyrin on the amount of glycogen in the body. Experiments on guinea-pigs showed after large doses of antipyrin the liver contained one-fifth more glycogen per kilogram than in a similar series of animals not treated with antipyrin, and that the amount of sugar in the first series was also proportionately less. Since the same results were obtained with antifebrin, quinine, and sodium salicylate, they conclude that antipyretics appear to completely stop or to hinder the change of glycogen into sugar in the liver. It is to be remarked that the doses given to the animals were much larger proportionately than those given to man; but it is possible that repeated doses in man may produce the same effects as a single large dose in a guinea-pig, Savadovski found that 2 per centum antipyrin inhibits putrefactive decomposition, but it requires a 4 or 5 per centum solution for complete inhibition. In therapeutic doses the drug has no effect on the secretion of gastric juice, nor on stomach digestion. The drug acts as a sedative on the nervous system, but large doses produce convulsions. He could detect no effect on the sensory or motor nerves; but Blumenau has shown that tactile sensibility is greatly increased by the local action of the drug, while the sense of pain is diminished; and this has been found to be true clinically. In Savadovski's opinion the vomiting caused by antipyrin is of central origin.

While Maragliano and Bettelheim state that the fall of internal temperature goes *pari passu* with the rise of the skin-temperature, which is due to dilatation of the cutaneous vessels, according to Savadovski the fall of internal temperature may occur without dilatation of the skin vessels. In regard to the action of the drug on the heat-centers of the brain, the conclusion reached was that antipyrin acts as an antipyretic first by increasing the loss of heat and acting on the vasomotive thermic center, which lies in the front part of the corpus striatum; and that it acts also on the trophic center in the posterior part of the corpus striatum.

On account of its action on the nervous system antipyrin has been used in many nervous diseases. Germain Sée has recommended it hypodermically for pain, and his results have been more or less confirmed by other observers. Sée has had good results also from its use hypodermically in rheumatism, in the pains of chronic gout and rheumatism, and in those of locomotor ataxia. The drug is now a recognized remedy in many forms of functional headache and neuralgia. It has been used in chorea with success, in cases of epilepsy in which the convulsions were associated with menstruation, and in migraine following epilepsy. Sonnenberger and Griffith have obtained good results from its use in pertussis; it should be given in the early stages of the disease. A case of diabetes insipidus has been reported in which the drug was of great service, reducing the amount of urine from six or ten to two litres a day. The dangers and drawbacks of the drug have been dwelt upon at such length in recent literature that they need not be mentioned here.

Salicylic acid has received a good deal of attention in the last year. Huber has shown that it is one of the safest and most important diuretics that we have; the experiments and results of Haig were mentioned at length in the January number of THE JOURNAL AND EXAMINER. The greatest increase in the amount of urine occurs in rheumatic fever and serous pleurisy, whether the temperature be raised or not. The value of Ehring's salicylate of bismuth has been mentioned in a recent number of THE JOURNAL AND EXAMINER. It is to be recommended in disorders of the stomach and intestines in children, and should be given with glycerine and water in a 5 per centum mixture. It should not be given in powder.

Förster, in investigating the effect of alcohol in fasting men on the excretion of phosphoric acid by the urine (absolute alcohol, of 30 to 50 per centum strength), found that the amount of phosphoric acid excreted was greater after alcohol, and was the greater the more pronounced were the symptoms produced by the alcohol. He thinks that these results hold good for the sick as well as for the healthy.

The action of chloroform as an antiseptic had been investigated by Salkovski. He found that 5 cc. dissolve completely in one litre of water at the temperature of the room, and that this solution is antiseptic and disinfectant, preventing the growth of bacteria. It will keep milk for months, and urine and pathological effusions for a long time. It has no action on organic ferments, such as those of digestion; but it kills the bacillus of anthrax in an hour and a half, though the spores are not affected; it kills cholera bacilli in one minute.

Aldehyde has been investigated by Albertoni and Pisenti. In the vessels of the different organs it produces a condition of arterio-sclerosis, and various degrees of fibrosis are produced, and an interstitial growth of fibrous tissue in the liver, with an infiltration of round cells, which is a true commencing cirrhosis. The mucous membrane of the stomach is but slightly affected, and the kidneys sometimes slightly, sometimes greatly affected, the glomeruli being filled with blood.

In a series of investigations Mosso has filled many gaps in our former knowledge of the action of cocaine, but his results have been already extensively published. He recommends cocaine as one of the best stimulants both in ordinary conditions and in narcosis from poisons. It is antagonistic to chloral hydrate, though it has no effect on the fall of temperature produced by chloral. It is also an antagonist to ether and chloroform, and these may be inhaled in acute cocaine poisoning to prevent tetanus of the respiratory muscles; when the first danger is over, chloral should be given. Artificial respiration should be performed if the breathing stop entirely. Nitrite of amyl is not antagonistic to cocaine, as has been asserted.

Methylal has been used as an hypnotic in various diseases. It produces sleep in chronic excitable conditions, senile dementia, and progressive paralysis, but it is useless in mental excitement due to alcohol, and in recent mental disease. It has no hypnotic effect when given subcutaneously.

Fröhner has shown that the prolonged use of paraldehyde produces effects similar to those of alcohol; it may produce methæmoglobinæmia, mathæmoglobinuria, and albuminuria.

Amylene hydrate, which has been recommended as an hypnotic, produces sleep in nervousness, excitability, paralysis, delirium tremens, senile decay, convalesence, anæmia, phthisis, and in febrile patients. It is of no use in insomnia due to pain. The drug stands between chloral and paraldehyde, fifteen grains of chloral being equal to thirty of amylene hydrate, or forty-five of paraldehyde. It has proved useful in chronic alcoholism, and in morphinism and encephalomalacia.

Cramer has found sulfonal useful in a large percentage of insane patients. Others have found it useful as an hypnotic.

Rosenbach has recommended ergot in the treatment of cardiac disease, with the view of increasing the activity of the walls of the blood-vessels, and of augmenting the pressure in the arterial system. It is useful in aortic regurgitation, in "idiopathic" dilatation, and in cases of arterio-sclerosis. It regulates the pulse, diminishes dyspnœa and palpitation, but has no effect on the œdema. It may be given in small doses every two or three hours.

Haslund has published some interesting results on the treatment of psoriasis with large doses of iodide of potassium. He prescribes at first a 5 per centum solution, half an ounce being taken four times a day. In two days another half an ounce is added to the dose, until the patient is taking six ounces daily. The strength is now increased to 6 per centum, thirty grains being added to the daily dose. Small children begin with a 2.5 per centum solution, and advance to the minimum dose for the adult. When the patient reaches the dose of ten drachms daily the effects must be watched with care. Of the fifty cases treated, forty were completely cured, four were improved, and in six there was no result. The duration of treatment was between two and a half and fifteen weeks, and the dose used during the course of treatment was between 40 and 280 drachms. The large doses were well borne, and there was but little iodism. The patients increased in weight during the treatment. It was noticed that the pulse was greatly increased in frequency, up to 100, 130, and even 140. The number and size of the blood-corpuscles was unaffected, and there was no diminution of the amount of fat, no atrophy of glands.

